# Estimation of Periodontal Inflamed Surface Area by Salivary Lactate Dehydrogenase Level Using a Test Kit

**DOI:** 10.3390/jcm13195904

**Published:** 2024-10-03

**Authors:** Koichiro Irie, Satsuki Sato, Yohei Kamata, Yuki Mochida, Takahisa Hirata, Motohiro Komaki, Tatsuo Yamamoto

**Affiliations:** 1 Department of Preventive Dentistry and Dental Public Health, Kanagawa Dental University, Yokosuka 238-8580, Kanagawa, Japan; irie@kdu.ac.jp (K.I.); mochida@kdu.ac.jp (Y.M.); 2 Department of Periodontology, Kanagawa Dental University, Yokosuka 238-8580, Kanagawa, Japan; sato.satsuki@kdu.ac.jp (S.S.); kamata@kdu.ac.jp (Y.K.); t.hirata@kdu.ac.jp (T.H.); m.komaki@kdu.ac.jp (M.K.)

**Keywords:** periodontal diseases, lactate dehydrogenases, saliva, mass screening, cross-sectional studies

## Abstract

**Background:** Salivary lactate dehydrogenase (LD) levels are a feasible and useful parameter for screening periodontal diseases. The periodontal inflamed surface area (PISA) is useful to clinically assess periodontal diseases. However, PISA is difficult to calculate and PISA-compatible screening kits are required. We aimed to investigate the association between salivary LD levels, using a test kit, and PISA and PISA-Japanese and determine the feasibility and reliability of the salivary LD test kit for evaluation of periodontal status. **Methods:** This study included 110 patients (66.4% female, median and 25–75 percentiles of age were 66.5 and 53.0–75.0 years, respectively) who visited the Dental University Clinic in Japan. Resting saliva samples were collected from each participant and LD levels were evaluated in real time using a kit featuring an integer scale ranging from 1 to 10. PISA and PISA-Japanese were calculated using periodontal parameters. **Results:** The median salivary LD level was 4.0. The medians of PISA and PISA-Japanese were 46.9 and 61.0, respectively. Salivary LD levels were positively correlated with the bleeding on probing rate (*r* = 0.626, *p* < 0.001), PISA (r = 0.560, *p* < 0.001), and PISA-Japanese (*r* = 0.581, *p* < 0.001). **Conclusions:** Our results suggest that salivary LD levels assessed using the salivary LD kit showed a significantly positive correlation with PISA and PISA-Japanese. In addition, we developed the PISA estimation formula using salivary LD levels measured with a test kit, sex, and age.

## 1. Introduction

Periodontitis, one of the most prevalent oral diseases, is a chronic inflammatory disease that affects tooth-supporting structures, ultimately leading to tooth loss and contributing to systemic inflammation [1]. Traditionally, the diagnosis of periodontitis is based on clinical examinations and the evaluation of periodontal parameters, including bleeding on probing (BOP), probing pocket depth (PPD), and clinical attachment level (CAL) [2]. In the screening for periodontal disease, the Community Periodontal Index (CPI) is widely used to adapt these clinical indicators to representative teeth [3]. However, clinical examinations using a periodontal probe are often accompanied by pain and variations in measurements, which can be influenced by the skill of the operator [4,5]. Therefore, for the screening of periodontal disease, non-invasive and simple methods not requiring direct examination of periodontal tissue by a skilled professional, such as salivary examination, have been considered [6,7].

Salivary enzymes have been proposed as candidates for disease markers of periodontitis [6,8]. The host response to periodontitis includes the production of various enzymes, which are released due to the damage and death of stromal, epithelial, or inflammatory cells [9]. Previous studies have investigated the association between aspartate aminotransferase (AST), alanine aminotransferase (ALT), lactate dehydrogenase (LD), and alkaline phosphatase (ALP)—enzymes commonly used in blood tests for systemic disease screening—and the condition of periodontal tissue [10,11]. Studies have shown that salivary LD levels in patients reflect their periodontal status [10,11,12,13,14,15], and that salivary LD levels are a feasible and useful parameter for screening periodontal diseases [10,12,13,14,15]. LD is present in the cytoplasm of nearly all human cells, and its extracellular presence in saliva may be associated with periodontal tissue breakdown [10].

Recently, a new and simple commercially available test kit has been developed to assess salivary LD levels and its association with periodontal disease has been reported [16,17]. The kit does not require saliva samples to be sent to a laboratory; instead, saliva is applied to the test paper and LD activity can be determined chairside within 60 s. This allows for real-time results, enabling immediate explanation and health guidance right after the test. Unlike the time-consuming CPI screening, this kit is used in group dental checkups at workplaces to reduce screening time [18]. Therefore, the use of this kit, along with its broader application, is anticipated.

The periodontal inflamed surface area (PISA) has been proposed as a measure to assess the overall periodontal inflammatory status in patients with periodontitis [19]. PISA is calculated using BOP, along with either PPD or CAL and gingival recession, to quantify the pocket epithelial surface area with bleeding, measured in square millimeters, for all teeth. It quantifies the inflammatory burden of periodontitis by measuring the extent of inflamed periodontal tissue. PISA is a valuable index for both clinical and epidemiological assessments, as it represents the inflammation status of patients in a single continuous variable. Consequently, PISA serves as a useful tool for dentists to communicate periodontal disease information with medical doctors. However, the calculation of PISA is complex, requiring probing pocket measurements of all teeth, as well as BOP and PPD data. This complexity highlights the need for screening kits that are compatible with PISA.

The association between salivary LD levels and periodontal conditions such as BOP and PPD has been examined using test kits in young adults, i.e., university students [15,16]. However, the association between salivary LD levels and the PISA in middle-aged and older adults remains unclear. Recently, acknowledging the racial diversity in root morphologies, the Japanese version of PISA (PISA-Japanese) was developed specifically for Japanese populations [20]. Therefore, in this study, we aimed to investigate the association between salivary LD levels, using a test kit, and PISA and PISA-Japanese and determine the feasibility and reliability of the salivary LD test kit for assessing periodontal status in Japanese adults. Furthermore, we examine the PISA estimation formula using salivary LD levels with test kits that can assess the condition of periodontal tissue without periodontal probing.

## 2. Materials and Methods

### 2.1. Study Participants

The present study included 110 patients who visited the Kanagawa Dental University Yokohama Clinic in Japan between June 2023 and December 2023. Patients visited the clinic for dental treatments. Inclusion criteria were age ≥20 years, ≥5 teeth, non-smoker or past smoker, no use of antimicrobials within three months, and no mouth ulcers, wounds, or acute inflammation of the mouth on the day of examination. Current smokers were excluded due to the known impact of smoking habits on LD levels [21].

### 2.2. Measurement of Salivary LD Level

**Figure 1:**
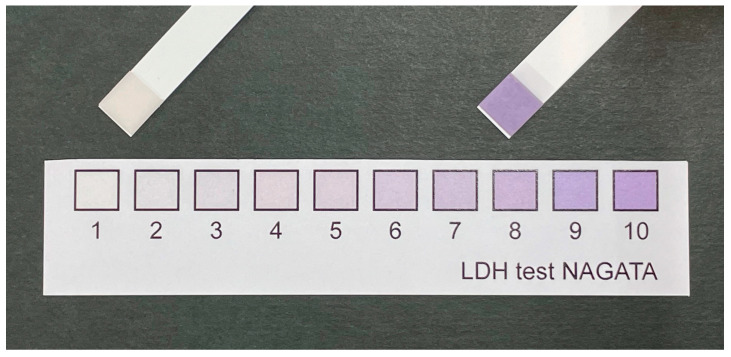
Scale guide and a representative sample. The salivary LD test kit includes a color-changing sheet (Lot CC2) that uses an integer scale from 1 to 10 to indicate the LD level.

Salivary LD levels were measured using a kit (LDH Test NAGATA; Nagata Sangyo Co., Shiso, Japan). The kit consists of reagent strips containing 3.347 mg/mL nicotinamide adenine dinucleotide, 500 U/mL diaphorase, 5.0 mg/mL nitroblue tetrazolium, 12 mg/mL Tris buffer, 40 mg/mL lithium lactate, and 10 mg/mL bovine serum albumin. In the presence of LD, formazan (purple) is produced from nitroblue tetrazolium (faint yellow) [16,17]. Before the oral examination, a resting whole saliva sample (approximately 0.5 mL) was collected from each participant and immediately applied to a reagent strip, following the manufacturer’s protocol. This led to a color change, indicating the presence of LD. The level of LD was recorded after 60 s, according to the kit’s scale guide (Lot CC2) (scale, 1–10) (Figure 1). Each dentist, trained by a single dentist (K. I.), evaluated the color change and determined the corresponding color value.

### 2.3. Oral Examination

After measuring LD levels, two trained dentists (S.S. and K.Y) examined the participants’ oral health status. They counted the number of teeth present and measured PPD and BOP at six points per tooth for all teeth using a manual probe (PCP-UNC 15; Hu-Friedy, Chicago, IL, USA). Based on the periodontal parameters, PISA and the PISA-Japanese version were calculated as previously described [19,20].

### 2.4. Questionnaire

The questionnaire included items on age, sex, smoking status, current medications, and general health, all assessed through patient interviews.

### 2.5. Ethical Approval

Written informed consent for all data used in the analysis was obtained from all participants, in accordance with the Ethical Guidelines for Medical and Biological Research Involving Human Subjects. The study protocol was approved by the Ethics Committee of Kanagawa Dental University (approval no. 967, 8 November 2023). This study was conducted in accordance with the principles of the Declaration of Helsinki.

### 2.6. Statistical Analysis

Descriptive statistics were employed to characterize the study population. The measurements and calculated values were presented as medians and interquartile ranges because the values were not normally distributed. Spearman’s rank correlation was used to examine the correlation between salivary LD levels and clinical variables. Multiple linear regression analyses were conducted to identify independent predictors of salivary LD levels. In addition, multiple linear regression analyses were performed to estimate PISA with salivary LD levels and other variables. BOP rate, PISA, and PISA-Japanese were analyzed separately to avoid multicollinearity because there was a strong correlation between the variables. The independent variables included sex, age, past smoking, medication, and number of teeth present. The sample size was calculated for linear regression, with hypothesized values of the population multiple partial correlation set at 0.36, a total of five predictors in the model, and five test predictors. Based on our calculations, the inclusion of 92 participants was deemed necessary to achieve a power of 80% with a two-sided significance level of 5%. The level of statistical significance was set at *p* < 0.05. Statistical analyses were conducted using SPSS version 29.0 (IBM Japan, Tokyo, Japan).

## 3. Results

The study population comprised 37 male (33.6%) and 73 female (66.4%) patients, with a median (25 and 75 percentiles) age of 66.5 (53.0–75.0) years (Table 1). The mode and median of salivary LD levels were 3.0 and 4.0, respectively. The medians of PISA and PISA-Japanese were 46.9 and 61.0, respectively.

The correlation between salivary LD levels and other variables is shown in Table 2. Salivary LD levels showed a positive and significant correlation with BOP sites (*r* = 0.614, *p* < 0.001), BOP rate (*r* = 0.626, *p* < 0.001), mean PPD (*r* = 0.254, *p* = 0.008), PPD ≥ 4 mm sites (*r* = 0.257, *p* = 0.007), PPD ≥ 4 mm rate (*r* = 0.258, *p* = 0.008), PD ≥ 6 mm sites (*r* = 0.302, *p* = 0.001), PD ≥ 6 mm rate (*r* = 0.297, *p* = 0.002), PISA (*r* = 0.560, *p* < 0.001), and PISA-Japanese (*r* = 0.581, *p* < 0.001). Conversely, salivary LD levels were negatively and significantly correlated with the number of teeth present (*r* = −0.212, *p* = 0.026).

**Figure 2:**
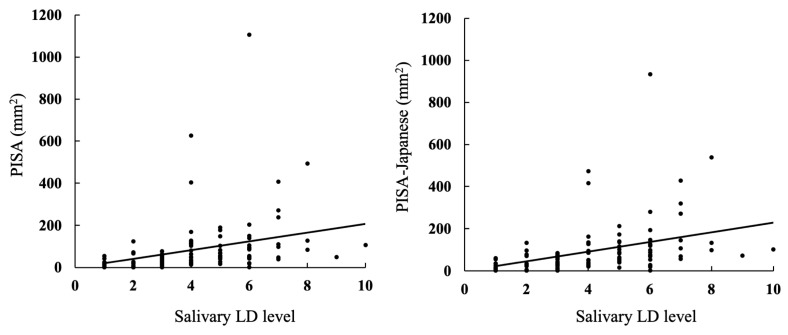
Scatter plots of PISA and PISA-Japanese against salivary LD level. The dots represent the data for each patient, while the lines indicate the regression trend.

The scatter plots for the PISA and PISA-Japanese against the salivary LD levels are provided in Figure 2. Most of the PISA values in the present study were less than 200 mm^2^.

The results of the multiple linear regression analysis with salivary LD levels as the dependent variable are shown in Table 3. In Model 1, the BOP rate was significantly associated with salivary LD levels (standardized β = 0.407, *p* < 0.001). In Models 2 and 3, PISA (standard β = 0.344, *p* < 0.001) and PISA-Japanese (standard β = 0.415, *p* < 0.001) were both significantly associated with salivary LD levels. In Models 2 and 3, the number of teeth present remained as an independent variable, but was not significant.

The results of the multiple linear regression to estimate PISA with salivary LD levels and other variables are shown in Table 4. In Models 1 and 2, PISA and PISA-Japanese were estimated with salivary LD levels, sex, and age.

Based on the linear regression equation, a predictive model was developed to estimate PISA and PISA-Japanese.

PISA = 22.787 × (salivary LD level: from 1 to 10) + 57.554 × (male: 1, female: 0) − 1.707 × (age in years old) + 80.125.

PISA-Japanese = 25.068 × (salivary LD level: from 1 to 10) + 47.007 × (male: 1, female: 0) − 1.833 × (age in years old) + 91.707.

## 4. Discussion

In the present study, we assessed the association between salivary LD levels, using an LD test kit (LDH Test NAGATA; Nagata Sangyo Co., Shiso, Japan), and PISA and PISA-Japanese in Japanese adults. Our results showed that the salivary LD levels using a test kit showed a significant positive correlation with both PISA and PISA-Japanese. Furthermore, salivary LD levels were also positively correlated with clinical parameters such as BOP and PPD. These results are consistent with those of previous studies that suggested salivary LD levels as a feasible and useful parameter for screening periodontal disease [10,12,13,14,15,16,17]. Notably, this study is the first to elucidate the compatibility of salivary LD levels using a test kit with PISA and PISA-Japanese.

Both Spearman’s rank correlation and the multiple linear regression analyses showed that salivary LD levels were positively correlated with BOP rate, PISA, and PISA-Japanese and that the values of the correlation coefficients and standardized coefficients β for BOP rate were higher than those for PISA and PISA-Japanese. BOP rate was more strongly correlated with salivary LD levels because while PISA is the area of inflammation [19], BOP rate is the percentage of inflammation and indicates the progression of periodontal disease. However, LD levels in saliva reflect the magnitude of tissue destruction in a single oral cavity. It is thought that the higher the rate of gingival inflammation, the more intense the gingival tissue destruction and the higher the LD activity in saliva.

PISA-Japanese was more highly correlated with salivary LD levels than PISA. The difference was primarily attributed to variations in the tooth-level PISA of the maxillary molars and the mandibular second molar. Molar teeth have larger root surface areas than any other tooth type, and racial differences have been observed in molars [22]. Therefore, the number of roots is positively correlated with the root surface area, and this morphological difference is consistent with the results of this study. The results of the present study, which showed that PISA-Japanese was more appropriate than PISA for Japanese people, were consistent with the results of previous studies that compared PISA with body mass index [20].

In this study, we developed the PISA estimation formula using salivary LD levels measured with a test kit, sex, and age, without a probing pocket. For example, from our PISA estimation formula, the PISA at the age of 40 years was 160.55 mm^2^ with an LD value of 4. According to previous studies, a cutoff value of PISA ≥ 130.33 mm^2^ could be a strong predictor of the presence of periodontitis [23]. In previous studies, the cutoff value for the LD activity to distinguish between gingivitis and a CPI score of three or higher ranged from 0.27 U/mL to 0.38 U/mL [10,12,14,15], while the LD activity of a score of four measured by the kit that we used was 0.36 U/mL. Therefore, based on these findings, this estimation formula using salivary LD measured with a test kit may be useful for estimating PISA.

PISA requires a probing periodontal pocket, which induces pain and poses a risk of bacterial infection for patients with periodontal inflammation [4]. Additionally, PISA assessments require the expertise of a well-trained dentist. In contrast, this salivary kit can obtain results within 60 s, making it easy for anyone to measure. Therefore, in the future, if medical doctors can measure the LD levels in saliva in patients with diabetes and deduce the PISA from the results, they may refer patients from medicine to dentistry.

However, further studies are needed to validate the formula for predicting PISA because the coefficients of determination of the linear regression models were relatively low (0.17 and 0.22). One reason for these low values was the small sample size. In the sample size calculation of the linear regression, the hypothesized value of the population multiple partial correlation was set at 0.36. Another reason may be that our study population primarily consisted of patients with stable periodontal status. The median of PISA was 46.9 (19.4–95.0) in the present study. Past studies showed that severe periodontitis group values ranged from 934.71 to 3274.96 mm^2^; the moderate periodontitis group ranged from 521.58 to 790.30 mm^2^; and the mild periodontitis group values ranged from 110.16 to 447.01 mm^2^ [23].

In further studies, current smokers must be included. In the present study, current smokers were established as an exclusion criterion to eliminate the effects of smoking, which may influence salivary LD levels [21], thereby reducing the sample size. Although past smokers (8.2%) were included in this study, multiple regression analysis revealed no significant correlation with salivary LD levels or PISA. This study did not provide information on the abstinence period of past smokers, and this limitation should be addressed in future research.

A limitation of this study was the small number of subjects with diabetes, comprising only 5.5% of the total participants. A higher percentage of individuals with diabetes have periodontitis compared to those without diabetes [24]. Additionally, it has been reported that individuals with diabetes exhibit higher salivary LD activity than those without diabetes [25]. Further studies are needed to confirm the effectiveness of the salivary LD kit in subjects with diabetes mellitus, as well as those with severe periodontal conditions.

In this study, only one type of biomarker, salivary LD levels, was utilized for estimating PISA, primarily to reduce testing costs. However, the use of fewer biomarker types may compromise screening accuracy. To enhance the accuracy of our estimates, we employed a multiple regression analysis model incorporating information obtained from the questionnaire. Screening for periodontal disease using multiple biomarkers, as demonstrated in previous studies [14], should also be considered.

Our study had other limitations. All the patients were recruited from a university clinic since the participants were not community-dwelling adults. Therefore, large-scale studies involving the general population are required to confirm our results. Moreover, this study had a cross-sectional design. Repeatedly high LD levels may be an indicator of disease progression, which requires confirmation through a prospective cohort study. It should also be considered whether salivary LD levels can predict the progression of periodontal disease, as demonstrated in previous studies [26].

## 5. Conclusions

Salivary LD levels assessed using the salivary LD kit showed a significantly positive correlation with PISA and PISA-Japanese. For Japanese people, PISA-Japanese correlated better than PISA with salivary LD levels using the salivary LD kit. In addition, we developed the PISA estimation formula using salivary LD levels measured with a test kit, sex, and age.

## Figures and Tables

**Table 1:** Characteristics of participants.

Variable	Total (*n* = 110)
Sex	
Male	37 (33.6%)
Female	73 (66.4%)
Age (years)	66.5 (53.0–75.0)
Past smoking	9 (8.2%)
Diabetes mellitus	6 (5.5%)
Medication	41 (37.3%)
Number of teeth present	26.0 (22.0–28.0)
Salivary LD level	
1	10 (9.1%)
2	15 (13.6%)
3	22 (20.0%)
4	19 (17.3%)
5	15 (13.6%)
6	17 (15.5%)
7	7 (6.4%)
8	3 (2.7%)
9	1 (0.9%)
10	1 (0.9%)
BOP sites	5.0 (2.0–9.0)
BOP rate (%)	3.9 (1.8–6.4)
Mean PPD (mm)	2.1 (1.9–2.4)
PPD ≥ 4 mm sites	3.0 (0–10.3)
PPD ≥ 4 mm rate (%)	2.4 (0–7.6)
PPD ≥ 6 mm sites	0 (0–1.0)
PPD ≥ 6 mm rate (%)	0 (0–1.2)
PESA (mm^2^)	974.0 (801.5–1168.0)
PESA-Japanese (mm^2^)	1164.9 (882.3–1329.5)
PISA (mm^2^)	46.9 (19.4–95.0)
PISA-Japanese (mm^2^)	61.0 (24.8–116.1)

Data are expressed as numbers (%) or medians (25–75 percentiles). BOP: bleeding on probing, LD: lactate dehydrogenase, PESA: periodontal epithelial surface area, PISA: periodontal inflamed surface area, PPD: probing pocket depth.

**Table 2:** Spearman’s rank correlation analysis for salivary LD level.

Variable	*r*	*p* Value
Age (years)	−0.011	0.912
Number of teeth present	−0.212	0.026
BOP sites	0.614	<0.001
BOP rate (%)	0.626	<0.001
Mean PPD (mm)	0.254	0.008
PPD ≥ 4 mm sites	0.257	0.007
PPD ≥ 4 mm rate (%)	0.258	0.007
PPD ≥ 6 mm sites	0.302	0.001
PPD ≥ 6 mm rate (%)	0.297	0.002
PESA (mm^2^)	0.122	0.202
PESA-Japanese (mm^2^)	0.062	0.522
PISA (mm^2^)	0.560	<0.001
PISA-Japanese (mm^2^)	0.581	<0.001

BOP: bleeding on probing, LD: lactate dehydrogenase, PESA: periodontal epithelial surface area, PISA: periodontal inflamed surface area, PPD: probing pocket depth.

**Table 3:** Factors associated with salivary LD level by multiple regression analysis with stepwise variable selection.

Model	Unstandardized Coefficients (B)	95% CI for B		Standardized Coefficients β	*p* Value
Dependent Variables		Lower	Upper		
Model 1					
BOP rate	0.118	0.068	0.169	0.407	<0.001
Model 2					
Number of teeth present	−0.057	−0.122	0.008	−0.158	0.083
PISA	0.005	0.002	0.007	0.344	<0.001
Model 3					
Number of teeth present	−0.061	−0.125	0.002	−0.168	0.056
PISA-Japanese	0.007	0.004	0.009	0.415	<0.001

BOP: bleeding on probing, CI: confidence interval, LD: lactate dehydrogenase, PISA: periodontal inflamed surface area. Model 1: independent variables entered into the model were sex, age, past smoking, medication, number of teeth present, and BOP rate. Model 2: independent variables entered into the model were sex, age, past smoking, medication, number of teeth present, and PISA. Model 3: independent variables entered into the model were sex, age, past smoking, medication, number of teeth present, and PISA-Japanese.

**Table 4:** Results of the multiple regression analysis with stepwise variable selection to estimate PISA or PISA-Japanese with salivary LD level and other variables.

Model	Independent Variables	Unstandardized Coefficients (B)	95% CI for B		Standardized Coefficients β	*p* Value	R^2^
Dependent Variables			Lower	Upper			
Model 1							
PISA	Salivary LD level	22.787	10.475	35.099	0.326	<0.001	0.166
	Sex (male)	57.554	5.526	109.582	0.199	0.030	
	Age	−1.707	−3.642	0.227	−0.157	0.083	
	Constant	80.125					
Model 2							
PISA-Japanese	Salivary LD level	25.068	14.234	35.901	0.395	<0.001	0.217
	Sex (men)	47.007	1.228	92.787	0.178	0.044	
	Age	−1.833	−3.535	−0.131	−0.186	0.035	
	Constant	91.707					

CI: confidence interval, LD: lactate dehydrogenase, PISA: periodontal inflamed surface area. Models 1 and 2: independent variables entered into each model were salivary LD level, sex, age, past smoking, medication, and number of teeth present.

## Data Availability

The data that support the findings of this study are available on request from the corresponding author. The data are not publicly available due to privacy or ethical restrictions.
